# Biopsychosocial approach to tendinopathy

**DOI:** 10.1136/bmjsem-2022-001326

**Published:** 2022-08-01

**Authors:** Nathan Edgar, Christopher Clifford, Seth O'Neill, Carles Pedret, Paul Kirwan, Neal L Millar

**Affiliations:** 1 Institute of Infection, Immunity and Inflammation College of Medicine, Veterinary and Life Sciences, University of Glasgow, Glasgow, UK; 2 Department of Physiotherapy, NHS Greater Glasgow and Clyde, Glasgow, UK; 3 Department of Physiotherapy, School of Allied Health Professionals, University of Leicester, Leicester, UK; 4 Sports Medicine and Imaging Department, Clinica Mapfre de Medicina del Tenis C/Muntaner, Barcelona, Spain; 5 Discipline of Physiotherapy, Trinity College Dublin School of Medicine, Dublin, Ireland

**Keywords:** tendinopathy, psychology, sociology

## Abstract

Tendinopathy describes a spectrum of changes that occur in damaged tendons, leading to pain and reduced function that remains extremely challenging for all clinicians. There is an increasing awareness of the influence that psychological and psychosocial components, such as self-efficacy and fear-avoidance, have on rehabilitation outcomes in musculoskeletal medicine. Although it is widely accepted that psychological/psychosocial factors exist in tendinopathy, there is currently a distinct lack of trials measuring how these factors affect clinical outcomes. Biopsychosocial treatments acknowledge and address the biological, psychological and social contributions to pain and disability are currently seen as the most efficacious approach to chronic pain. Addressing and modulating these factors are crucial in the pathway of personalised treatments in tendinopathy and offer a real opportunity to drive positive outcomes in patients. In this education review, we also provide the current evidence-based guidance on psychological and psychosocial developments in musculoskeletal medicine and how these may be translated to treating tendinopathy using a biopsychosocial model.

**Key messages**

**What is already known**

- There is currently a lack of clear functional pathways to account for clinical disease in tendinopathy.
- Persistent tendon pain can have a negative psychological impact on patients.
- There is an increasing awareness of the impact of these psychological components in facilitating or impeding rehabilitation outcomes.
- Evidence from other musculoskeletal (MSK) disorders suggests that there is therapeutic potential for understanding psychological factors in more detail.
- Addressing psychosocial factors can enhance the management of chronic conditions and improve outcomes.

**What are the new findings**

- In MSK settings, wider use of a biopsychosocial model in patient education would allow more scope for optimal management and recovery.
- Based on finding in other chronic MSK conditions, we suggest that reframing patient beliefs surrounding pain, treatment and misconceptions will reduce the impact that psychosocial factors have on rehabilitation outcomes.
- There is currently a distinct lack of robust trials measuring how psychosocial factors affect clinical outcomes in tendinopathy.
- Further research is required to determine how best to address psychosocial factors in the context of patient education.

## Introduction

Often disabling, painful and persistent, tendinopathy is characterised by activity-related pain and loss of function due to mechanical loading.^1^ While promising advances in basic and clinical science have resulted in new insights into the mechanisms that may drive disease; these have yet to be translated to the patients that sit in front of us at the clinic. While many therapeutic modalities are available, exercise and loading programmes remain the best evidence-based first-line management. As clinicians who treat tendinopathy from normal recreational individuals to the sporting elite, we realise the significant impact on normal and professional daily activities tendon disease can cause yet sometimes overlook that disability that can persist beyond 12 months. Accordingly, persistent tendon pain can have a negative psychological impact on patients,^2 3^ leading to poor outcomes and resulting in a chronic disease profile. The multidimensional pathophysiology surrounding tendinopathy is not new; however, there is increasing awareness of the influence that psychosocial and psychological components can have in facilitating or impeding rehabilitation outcomes.^4^ Our understanding of other musculoskeletal (MSK) disorders suggests therapeutic potential for understanding psychological/psychosocial factors in more detail.^5 6^ This education review discusses the current psychological, and psychosocial developments thought to play a role in MSK medicine and how these may be translated to treating tendinopathy through a biopsychosocial model.

## Biopsychosocial factors in chronic MSK conditions

In chronic MSK conditions, psychosocial factors such as fear, anxiety and depression have been shown to affect pain and disability levels, harming rehabilitation.^7 8^ Adverse psychosocial exposure, culminating in depression, stress or a sense of hopelessness, can exacerbate chronic conditions and contribute to suboptimal patient outcomes.^9^ In these chronic conditions, an individual’s perception of their ability to succeed in particular situations has influenced the relationship between pain and disability.^10–12^ This is described as self-efficacy; high self-efficacy is associated with lower levels of pain and disability and overall better physical functioning.^8^

In addition to self-efficacy, fear-avoidance beliefs have been shown to influence rehabilitation outcomes. The fear-avoidance model describes the interpretation of pain via maladaptive or adaptive pathways. It is commonly used to explain how psychological factors can influence the perception and development of chronic pain.^13^ Negative perceptions of pain can lead to a catastrophising response within the maladaptive pathway. The resulting hypervigilance and disuse can develop into kinesiophobia, beginning a harmful cycle of chronicity,^14 15^ which has recently been highlighted in Achilles tendinopathy.^16^ In addition, hypervigilance and avoidance of physical activity can cause deconditioning of the MSK system,^17^ predisposing to further injury.

One of the most commonly used instruments to identify fear-avoidance beliefs within MSK clinics is the Tampa Scale for Kinesiophobia (TSK).^18^ This self-reporting questionnaire aims to differentiate between non-excessive fear and phobia in patients with chronic MSK pain. It focuses on domains such as fear of movement, fear of physical activity, fear-avoidance and fear of reinjury and has already been used with varying results in Achilles tendinopathy.^19^ Other scales have been developed to provide a more comprehensive picture of pre-existing psychosocial beliefs. The ‘Fear Avoidance Components Scale’ was developed in 2016^20^ and combines components of several well-established scales, including the TSK. New scales also aim to provide a more complete depiction by subdividing specific populations. The ‘Athlete Fear Avoidance Questionnaire’ is a sport-specific scale that uses terms such as ‘I will never be able to play as I did before the injury’ to address psychological barriers specific to athlete populations that may have been overlooked in the past.^21^

## Psychosocial factors in tendinopathy

The structural changes seen on imaging of tendinopathic tendons often do not explain the response to exercise led interventions,^22 23^ suggesting that physical factors are not the only influential component of rehabilitation. In fact, psychological factors may exert more influence over clinical outcomes than visible structural damage.^24^ Indeed, psychological fears, patient rating of pain and tendinopathy’s impact on quality of life were all recently deemed core health domains in tendinopathy.^25^ Misconceptions regarding pain can lead to psychological distress, depression and increased sensitivity to pain,^26 27^ which can limit patient progression in loading programmes.^28^ For clinicians, understanding how psychosocial factors can affect tendinopathy is essential to educate patients on the possible influence these factors can have on the pain experienced. Addressing fear-avoidance beliefs about pain may improve outcomes as patients are made aware that not all pain experienced is harmful. In cases where psychosocial factors are harnessed effectively, they may have the potential to facilitate outcomes. Indeed, a patient’s level of self-efficacy may be a stronger predictor of non-surgical outcomes than the structural defects that exist in imaging.^29 30^

Dunn *et al* found a modest association between physiological issues and the clinical outcome in tendinopathy but acknowledged the need for more longitudinal studies to investigate the sway of psychosocial issues on clinical outcome.^29^ This is also voiced by Mc Auliffe *et al,* who accept that although this future research may not affect clinical outcomes, it would give greater clarity on how these psychological factors can influence prognosis and pain modulation in patients.^31^ Although it is widely accepted that psychosocial factors exist in tendinopathy, there is currently a distinct lack of trials measuring how these factors affect clinical outcomes. In addition to further trials, one must also ask how patients’ psychological outlook on their condition can be improved to drive positive outcomes.

## Patient education in tendinopathy

Patient education aims to provide the individual with a greater understanding of the condition that affects them.^32^ More recently, this knowledge acquisition has developed to allow patients to make informed choices about treatment and management. In chronic conditions such as tendinopathy, where loading programmes are the initial treatment, educating patients on managing their condition is essential in gaining optimal outcomes.^4^ This element can be referred to as health literacy, defined as patients having the ability to seek, understand and act on information relating to their health.^33 34^ The goal of successful patient education should always be patient empowerment, whereby the patient has the ability to largely self-manage their condition. However, self-management is not always successfully fostered by current healthcare systems.

Central to this approach is identifying and understanding key factors, such as self-efficacy and pain catastrophising, which have been shown to influence MSK health outcomes.^35^ Successful identification and management directed at these factors may increase patient empowerment, enhancing recovery. The responsibility of developing patients’ health literacy and understanding psychosocial factors lies with the clinician; we determine the parameters of the patient–clinician interaction, including communication style and being open to patient questions. A successful interaction between health professionals and patients, whereby the patient is involved in the consultation process and receives emotional support, can be described as a working alliance. This form of partnership is associated with adherence behaviours and improved outcomes in other chronic MSK conditions.^36^ It has received calls to be investigated further in tendinopathy.^4 37^

## What should a biopsychosocial approach involve?

**Figure 1:**
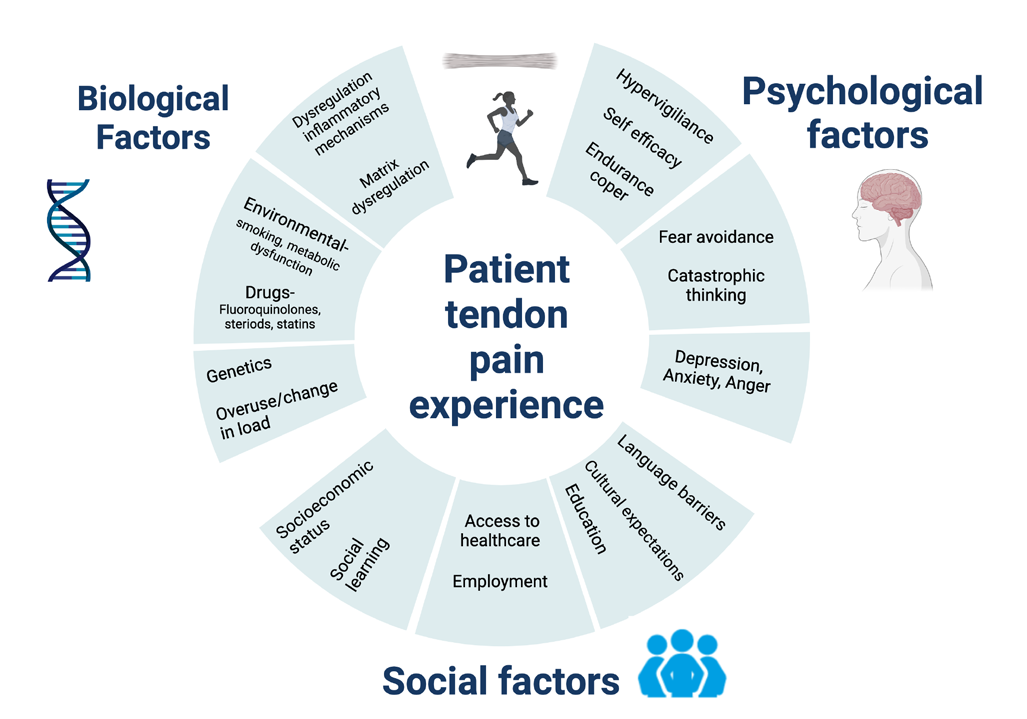
Biopsychosocial factors involved in tendinopathy. Core examples of the biological, social and psychological factors that should be considered when assessing a patient with tendinopathy.

In MSK settings, particularly in tendinopathy, we propose that wider use of a biopsychosocial model in patient education (figure 1) would allow more scope for optimal management and recovery, as evidenced from other chronic MSK conditions.^6 38 39^ As the successor to the biomedical model, this interdisciplinary approach acknowledges the interactions between biology, psychology and social factors.^40^ The varied clinical picture, high recurrence rates and persistent functional impairments that are often synonymous with tendinopathy suggest that there are more factors at play than merely physiology.^41^ Education on pain mechanisms and treatment options have largely been key in the biomedical model of patient education. To advance this further, the biopsychosocial model allows for psychological and psychosocial factors, which are often the cause of suboptimal rehabilitation outcomes, to be addressed.

## Reframing biopsychosocial factors through patient education

There remains little empirical research demonstrating how biopsychosocial issues can be modified by patient education in tendinopathy. We, therefore, suggest that reframing patient beliefs surrounding misconceptions of pain, treatment and prognosis will reduce the impact that psychological and psychosocial factors have on rehabilitation outcomes based on other experiences in chronic MSK condition. The next section expands on the current evidence on pain, treatment and prognosis education

### Pain education

Following sustained pressure to address the social and cognitive aspects of pain,^42 43^ the International Association for the Study of Pain (IASP) revised the definition for the first time since 1979. The definition now acknowledges that pain is ‘…influenced to varying degrees by biological, physiological and social factors’.^44^ In chronic pain conditions, there is frequently a disconnect between clinical presentation and pathology observed on imaging.^45^ This is no different in cases of tendinopathy, where there is currently a lack of clear functional pathways to account for the clinical disease.^1^

Where pain can be defined as physiological or pathological (including neuropathic), clinical findings in tendinopathy often straddle both. The chronic pathological pain commonly seen in tendinopathy has been associated with functional changes causing increased sensitisation within the central nervous system; this may describe the resistance to tissue-based treatments and the chronicity that commonly ensues. Central sensitisation refers to the increased responsiveness of the central nervous system and encompasses features such as altered sensory processing in the brain, malfunctioning of descending antinociceptive mechanisms, and increased activity of pain facilitatory pathways. Central sensitisation is frequently present in various chronic MSK pain disorders^46^ and has been shown to involve psychosocial elements in upper limb tendinopathies^47^; with evidence of many chronic pain conditions sharing several typical features,^48^ it is plausible that central sensitisation that occurs in other tendinopathies may also involve these factors.

Furthermore, neuronal regulation is thought to play a role in tendon homeostasis and the presence of neuropathic pain in chronic tendinopathies has been proposed.^49^ Neuropathic pain is a result of damage or disease affecting the somatosensory system. While the presence of neuropathic pain in chronic tendinopathy has been proposed, the prevalence of neuropathic pain has not yet been studied in detail in clinical populations with tendinopathy.^50^ However, a recent study suggests that presence of neuropathic pain is not associated with a worse clinical outcome in patients with Achilles tendinopathy^51^ highlighting that further research is required in this arena.

Accordingly, pain may vary dependent on the area of tendinopathy.^41 48 52^ In light of this, it is important to make patients aware that not all pain felt is harmful, and in tendinopathy, the presence of pain during rehabilitation is acceptable^53^ With this in mind, it may be possible to reframe patients’ perception of the pain felt.^17^

Self-efficacy may improve if individuals comprehend that the pain they experience is not likely to be causing further degeneration of the tendon and will not progress to rupture. Indeed, Moseley^54^ described that pain is not always a measure of the state of tissue pathology, and often, the relationship between pain and tissue damage becomes less predictable as pain persists. Reproducing pain through loading and resistance programmes while ensuring the patient is aware that the pain felt is not harmful may help address fear-avoidance and catastrophising beliefs.^17 55 56^ Therefore, adequate education may potentially modify psychosocial factors, allowing pain experienced to be reconceptualised. Smith *et al*^57^ found a small but significant benefit of short-term painful exercises over pain-free exercises in chronic MSK conditions. However, for this approach to be effective, a close working alliance is required to elicit patient understanding, identify barriers to implementation, and understand the patient’s acceptable pain response.^58^

### Treatment education

**Figure 2:**
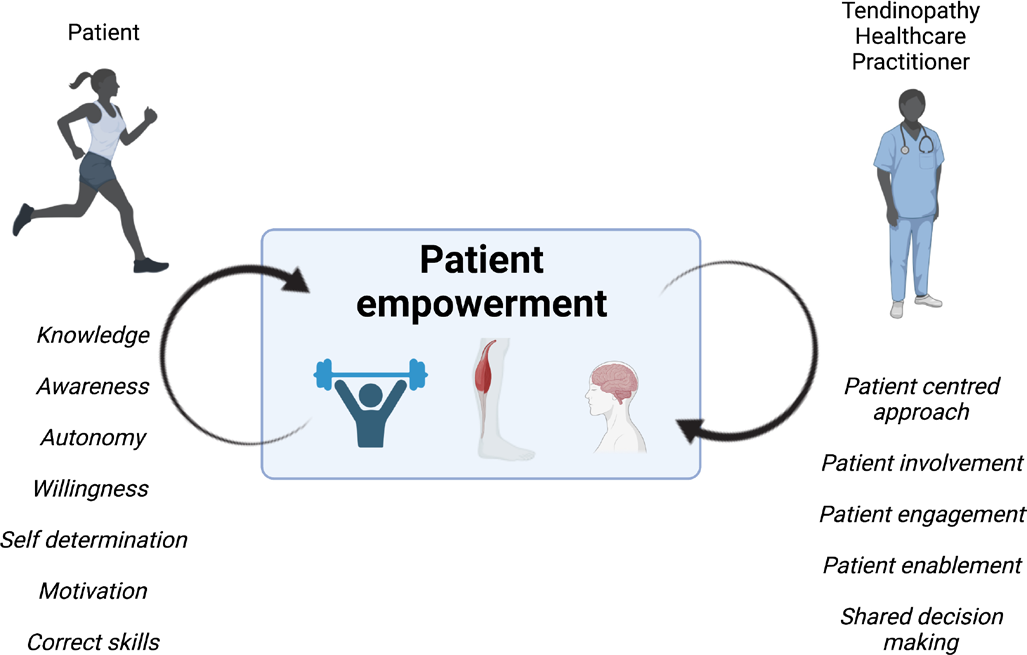
The role of patient empowerment in tendinopathy management. Examples of patient factors that can be addressed and discussed to better help the tendinopathy healthcare practitioner promote patient empowerment in the recovery journey.

Despite the large literature base surrounding the efficacy of these loading programmes, patient perception of them is generally poor.^59^ It is clear that merely prescribing exercise programmes and stating that adherence is necessary is not enough to increase self-efficacy and optimise outcomes. Indeed one of the most important problems in loading programmes for tendinopathy is that more than 50% of the patients abandon the programme, which further perpetuates chronicity. Patients must understand how the programme will benefit them, and barriers to adherence need to be broken down. Patient empowerment in tendinopathy was found to be key in facilitating adherence to treatment.^60^ Illustrating the importance of education further, Mellor *et al*^61^ concluded that in gluteal tendinopathy, education in combination with loading was a more effective treatment strategy than corticosteroid injections. This was further borne out in key studies in post-menopausal women.^62 63^ Patients should be made aware that despite often being referred to as an ‘overuse injury,’ self-prescribing rest as a self-management strategy will not elicit optimal outcomes. Through education, practitioners have the opportunity to improve patients’ self-efficacy (figure 2); this in itself may be more influential in optimising outcomes than any therapeutic treatment.

With a wide range of treatments existing claiming to treat tendinopathy with varying efficacy, it is equally important to guide patients to ensure they do not become over-reliant on passive treatments while neglecting active loading plans.^53^ In a time of patient autonomy, a balance must be struck to ensure that all decisions are made with an accurate understanding of their condition; education is the only way to enable this. Indeed the recent findings suggesting three stratified patient subgroups in Achilles tendinopathy^64^ (activity dominant, psychosocial dominant and structure dominant) further support the likely precision tendinopathy approach required in the biopsychosocial approach.

### Addressing misconceptions

**Figure 3:**
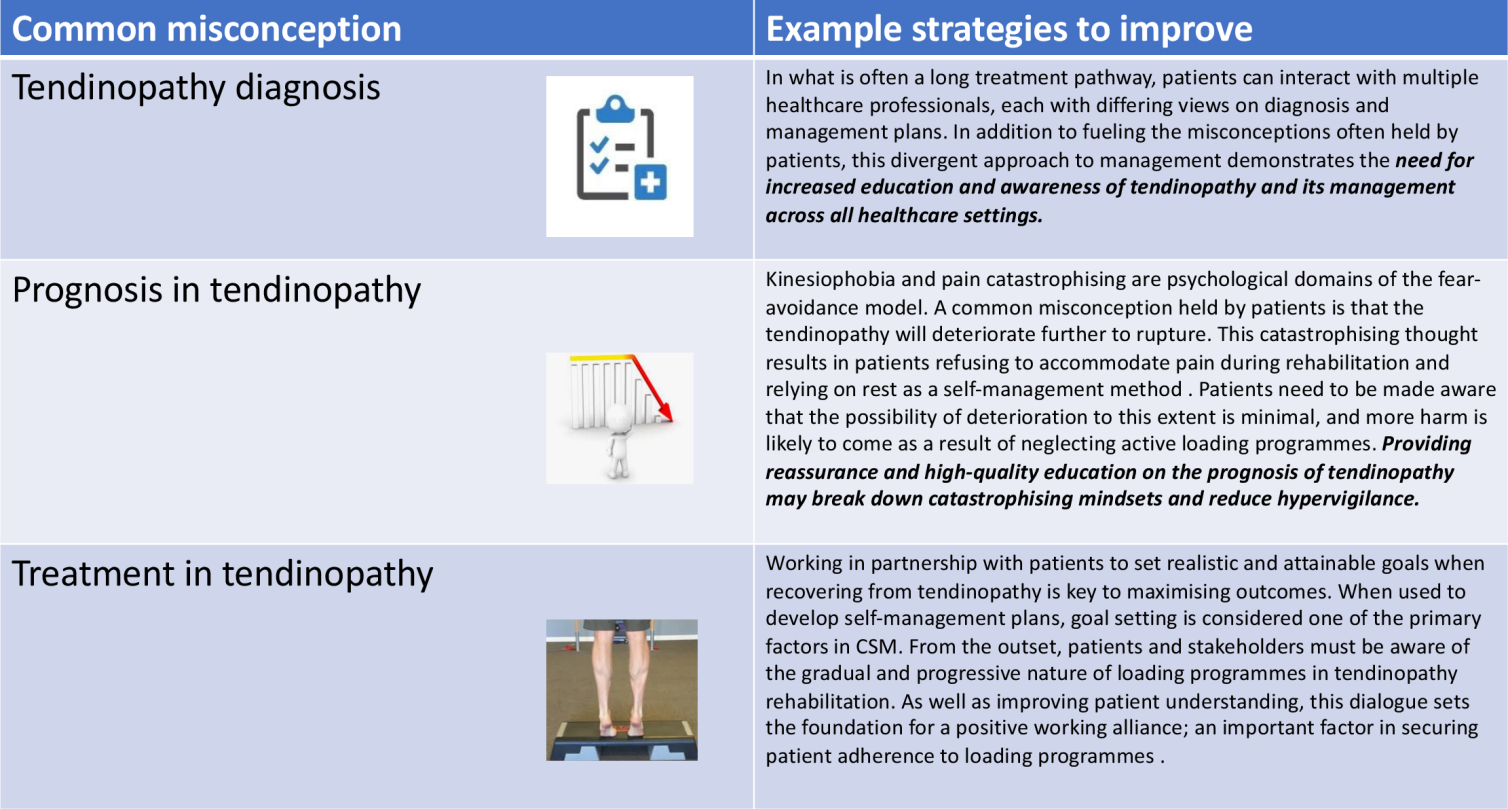
Misconceptions in tendinopathy diagnosis, prognosis and treatment and potential treatment strategies to address each aspect. CSM, Common-Sense Model of Self-Regulation.

The Common-Sense Model of Self-Regulation is a theoretical framework used to demonstrate how individuals act towards and manage threats to their health. When the threat is perceived, individuals develop both emotional and cognitive responses, which dictate how they respond to the threat.^65^ Through education and awareness of biopsychosocial factors, healthcare professionals have the opportunity to mould the patient’s perception of their condition, enhancing the outcome. However, due to the lack of definitive scientific grounding in tendinopathy, misconceptions in diagnosis, prognosis and treatment timeframe are common. These misconceptions must be addressed in a condition where the patient’s perception and degree of self-efficacy often influence clinical outcomes more than the existing structural damage (figure 3). Improving the patient’s health literacy stems from clear communication and being receptive to patient questions.

## Conclusion

In the largely chronic profile of tendinopathy, loading programmes remain the gold standard. However, psychological misconceptions are rife, and patient persistence with these programmes is instrumental in driving positive outcomes. Psychosocial factors such as fear-avoidance are not innate and are largely a result of social and environmental factors; therefore, they can be influenced through more targeted patient education. Psychosocial factors are increasingly being recognised as key components in tendinopathy; however, further research is required to determine how best to address them in the context of patient education and how to measure them in terms of transferable reported outcomes. Addressing and modulating psychosocial factors is crucial in the pathway of personalised treatments in tendinopathy and offers a real opportunity to drive positive outcomes.
